# Understanding rational non-adherence to medications. A discrete choice experiment in a community sample in Australia

**DOI:** 10.1186/1471-2296-13-61

**Published:** 2012-06-20

**Authors:** Tracey-Lea Laba, Jo-anne Brien, Stephen Jan

**Affiliations:** 1 Faculty of Pharmacy, University of Sydney, Camperdown, Sydney, Australia; 2 The George Institute for Global Health, Missenden Rd, Camperdown, Sydney, Australia; 3 Faculty of Medicine, University of New South Wales, Sydney, Australia

## Abstract

**Background:**

In spite of the potential impact upon population health and expenditure, interventions promoting medication adherence have been found to be of moderate effectiveness and cost effectiveness. Understanding the relative influence of factors affecting patient medication adherence decisions and the characteristics of individuals associated with variation in adherence will lead to a better understanding of how future interventions should be designed and targeted. This study aims to explore medication-taking decisions that may underpin intentional medication non-adherence behaviour amongst a community sample and the relative importance of medication specific factors and patient background characteristics contributing to those decisions.

**Methods:**

A discrete choice experiment conducted through a web-enabled online survey was used to estimate the relative importance of eight medication factors (immediate and long-term medication harms and benefits, cost, regimen, symptom severity, alcohol restrictions) on the preference to continue taking a medication. To reflect more closely what usually occurs in practice, non-disease specific medication and health terms were used to mimic decisions across multiple medications and conditions.161 general community participants, matching the national Australian census data (age, gender) were recruited through an online panel provider (participation rate: 10%) in 2010.

**Results:**

Six of the eight factors (i.e. immediate and long-term medication harms and benefits, cost, and regimen) had a significant influence on medication choice. Patient background characteristics did not improve the model. Respondents with private health insurance appeared less sensitive to cost then those without private health insurance. In general, health outcomes, framed as a side-effect, were found to have a greater influence over adherence than outcomes framed as therapeutic benefits.

**Conclusions:**

Medication-taking decisions are the subject of rational choices, influenced by the attributes of treatments and potentially amenable to intervention through education, strategic pricing and the altering of dosing characteristics. Understanding individual treatment preferences is thus an important step to improving adherence support provision in practice. Re-framing future interventions and policies to support rational and informed individual patient choices, is the way forward to realising the full potential health and economic benefits from the efficacious use of medications.

## Background

In developed countries, it is estimated that 50% of patients prescribed medications do not adhere to therapy [1]. Rates of non-adherence are estimated to be greater still within marginalised groups and in developing countries [1,2]. In spite of the potential to impact upon population health and expenditure [3], interventions to promote adherence have been shown, at best, to be of moderate effectiveness and cost effectiveness [4-6].

Recent evidence has indicated that a significant level of non-adherence is intentional involving deliberate decisions to adjust medication use [7-14]. Various factors have been individually associated with such medication-taking decisions including side effects, perceived drug effectiveness, and cost. However, the lack of a gold standard measure and indeed a lack of consensus on the definition of adherence have hindered any consistent and reliable set of conclusions to be drawn from the literature. Despite the recognition of the various factors that individually influence adherence, little is understood of their relative importance, their potential interactions and the extent to which individuals’ trade off one attribute of treatment for another in their decision-making.

Discrete Choice Experiment (DCE) is a survey methodology that can be used to elicit patient preferences to determine the relative influence of factors on decision making with regard to intentional medication adherence. Developed initially in marketing research, and now considered state of the art, DCEs have been used increasingly in health economics to elicit preferences for health services [15-18]. Recently, there have been two DCE studies that have explored and found a positive relationship between the impact of patient preferences for medication effectiveness and side effects on likely adherence in patients with bipolar disorder [19] and with diabetes [20]. In both studies, the effect of broader factors affecting adherence such as cost and treatment regimen were not included.

One other limitation of the DCE studies that have been conducted to date in this area is that they have been disease or regimen-specific. However with the possible exception of psychiatric conditions, there is some evidence to suggest that is an overly narrow perspective [5]. In practice, patients are likely to balance factors across multiple medications and across multiple conditions [11,12,21]. This study aims to explore medication-taking decisions that may underpin intentional medication non-adherence behaviour amongst a community sample and the relative importance of medication specific factors and patient background characteristics contributing to those decisions.

## Methods

A cross-sectional web-enabled survey was used.

### Survey instrument

The survey used a discrete choice experiment (DCE) to estimate the relative importance of medication factors on one’s decision to continue with a medication. The survey comprised three sections: the first contained questions regarding current medication use and implicit attitudes to medication using the *Beliefs About Medication Questionnaire (BMQ) – General*[22], the second, the DCE (10 hypothetical choice tasks), and the third, background information about the respondent.

### Instrument development

**Figure 1:**
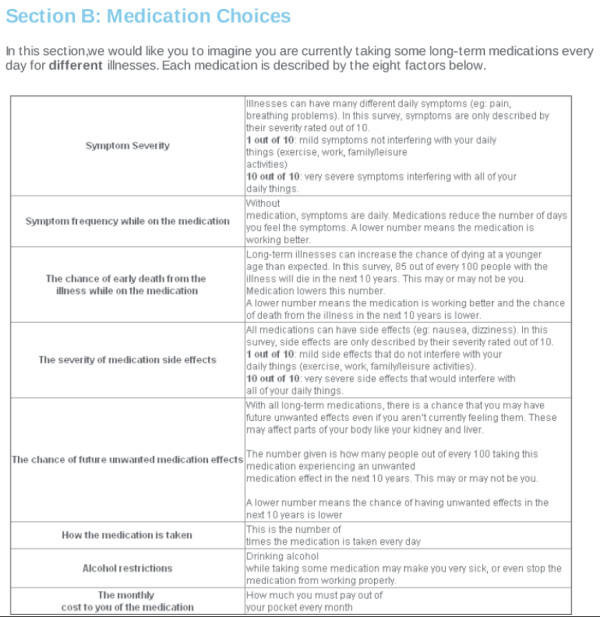
Description of factors provided to respondents.

In a DCE, respondents are offered a series of hypothetical pairwise alternatives (choice sets), and asked for each, to nominate the preferred alternative. Each alternative is described by a set of factors with pre-specified levels. The levels assigned to each alternative are varied successively across all choice sets. For this study, a set of factors covering four domains (i.e.: effect on current life, effect on future life, medication access, and ease of administration) were initially established through a systematic qualitative review of the qualitative and quantitative (observational) medication-taking literature published in English between 2000-May 2009 (Medline, Embase, PsychInfo and Cinahl) involving adult patients across all settings currently prescribed medications for chronic non-communicable diseases, and more specifically for cardiovascular disease (primary and secondary prevention). Studies only involving children/adolescents were excluded, as well as studies involving psychiatric, military or institutionalised patients to avoid the potential influence of psychosocial or institutional controls over adherence. Furthermore, quantitative studies that used surrogate measures of adherence (e.g. blood pressure) were excluded. The factors were further refined throughout survey pre-testing [15], amongst pharmacists and members of the general public to capture diverse educational, vocational, and medication-experience backgrounds. Eight factors considered most important through pre-testing and used in the final survey are summarised in Additional file 1. Additional file 1 also includes description of the levels of each factor. As the study was to be conducted within a general population with diverse medication experiences, extensive descriptions of each factor were presented in the survey preamble (see Figure 1). Furthermore, considering the diverse diseases, symptoms, medications and side effects possible, concepts of medication and health were described in non-disease specific terms.

The final survey included 10 choice sets. In each, respondents were presented with hypothetical long-term medication alternatives, ‘Medication A’ and ‘Medication B’. Respondents were asked to imagine they were currently taking both but to indicate which medication they would more prefer to continue to keep taking.

**Figure 2:**
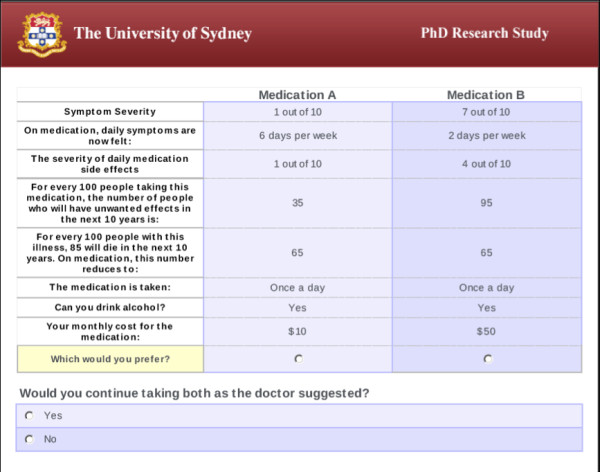
**Example choice question in the discrete choice experiment.** This figure is representative of one choice set. The levels of each factor changes from one choice set to the next.

On the basis of the exhaustive combination of factors and levels listed in Additional file 1, a statistically efficient design was generated using the choice experiment design software Ngene Version 1.0 [23]. The design was manually checked to ensure that each factor was balanced across the choice sets. The final experimental design consisted of 32 choice sets that were divided into 4 survey versions. Two additional choice sets were added to each survey version to check for consistency (a repeated choice set) and internal validity (a choice set with a dominated alternative according to *a priori* expectations – see Additional file 1). Rather than test for survey understanding, these tests are included to investigate whether responses are ‘rational’ according to the axioms of preference-based consumer theory [24]. Thus, each survey version comprised 10 choice sets. Each respondent was randomly assigned to one of four survey versions. An example choice set is presented in Figure 2. The survey was pilot tested (n = 18) to check for any problems with interpretation and face validity; only minor changes to layout were made.

### Participants

Based on the sample size calculations in Louviere et al [25], using the survey design characteristic, participants were recruited through Survey Sampling International, Australia (SSI), an online panel provider (minimum recommended sample size 48, without incorporating participant taste variability). Respondents on the SSI panel community are sourced non-probabilistically from all areas of the internet including SSI global panels, social media, and websites; respondent duplication or fraud is policed according to SSI policy. For this study, English-reading, Australian, adult (>18 years of age) panel members were invited to participate in the survey. The final sampling quota was requested to be representative of the national census data in terms of age and gender. Participants received entry into a competition conducted by SSI for prizes of value up to $AU2000. For participants completing the survey in less than 25 minutes, an opportunity to select a charity of which part of the income generated by SSI is donated was also given. An online participant information sheet was provided. Completion of the survey was considered as inferred consent.

### Analyses

Considering the vast number of factors affecting adherence across different types of medical conditions and treatments [26], a panel mixed multinomial (random parameters) logit (MMNL) model was used to analyse the choice data. This is a common method for analysing choice data in discrete choice experiments as it accounts for the panel-like structure of the repeated choice task and allows for a more general representation of variation in preferences between individuals [15,25]. This is in comparison to data segmentation via subgroup analyses, which requires *a priori* selection of the correct segmentation criteria and cut-offs that would account for statistically significant sources of preference heterogeneity.

This study investigated changes in utility (U) (i.e. preference to continue with a medication) when a level of a factor changes. A higher or more positive utility indicates increased preference to continue with a medication. The model assumed that the relationship of observed factor levels of each alternative and their corresponding weights is linear and corresponds to the following form:

(1) $$\begin{array}{l} U=\alpha +\beta_{1}*\left(current\;side\;effect\;severity\right)+\beta_{2}*(chance\;of\;future\;unwanted\;medication\\ effects)+\beta_{3}*\left(symptom\;frequency\;while\;on\;medication\right)+\beta_{4}*(chance\;of\;early\;death\\ from\;illness\;while\;on\;medication)+\beta_{5}*\left(out\;of\;pocket\;monthly\;cost\right)+\beta_{6}*(symptom\\ severity)+\beta_{7}*\left(regimen\right)+\beta_{8}*\left(alcohol\;restrictions\right)+\varepsilon_{\mathrm{isj}} \end{array}$$

where *U* is the utility or preference and *β*_*(1–8)*_ are the associated parameter estimates or relative weights for each factor. An alternative specific constant (α) was specified to capture left to right bias and to represent the mean of the distribution of the unexplained effects. The parameter estimates describe the magnitude of the utility change and indicate the relative impact of a unit change of a factor to the decision.

The choice model was estimated using NLOGIT Version 4.0. All the β parameters were considered linear, and initially treated as random with normal distribution with the exception of cost, which was initially treated as random with a constrained triangular distribution. Models were evaluated for goodness of fit using McFadden’s pseudo R^2^ and Akaike’s information criterion (AIC). The log-likelihood ratio test was used to determine the significance of a model against each other and to test for overall model significance compared to a model of the average utility (i.e. using the choice shares observed within the dataset). Final analyses were conducted using 1000 Halton or quasi-random draws from the random parameters distribution, which accelerates the estimation by a factor of 5–10 compared to simple pseudo-random draws [27].

The effect of patient background characteristics on the final model was investigated by forward stepwise addition. Backwards elimination of significant covariates was then performed. Additionally, it was hypothesised that there may be a differential parameter associated with the out-of-pocket cost factor. In Australia, the government subsidises the cost of medicine for most medical conditions through social health insurance (Medicare), with a co-payment for each prescription paid by consumers. People with a healthcare concession card (HC) are entitled to government-subsidised medications at a reduced co-payment rate. Private health insurance (PHI) is an optional medical insurance that may provide cover for the costs of medications that are not subsidised by Medicare. The Australian government provides income and age-related tax rebates and levies to increase the uptake of PHI. A differential out-of-pocket cost factor was therefore investigated based on household income level (INC), as well as healthcare concession card (HC) or private health insurance (PHI) through the incorporation of cost-factor interaction terms.

From the final model, the predicted choice outcomes for the sample as compared to the actual choice outcomes as they exist within the data were calculated. The relative importance of factors and their levels [17,28] were investigated.

The University of Sydney Human Research Ethics Committee approved this study (approval number 9-2009/12074).

## Results

Of the 1668 panel members initially invited to participate, 248 respondents began the survey, with 161 respondents (median age 57 years, 45% Male) completing the survey (participation rate 10% [29]). The participation rate indicates the efficiency of internet panels that use non-probability based sampling methods for participant selection and recruitment and in which response rate has no real meaning [29]. As such, the participation rate for this study reflects the expected rate for this online panel provider.

A summary of the characteristics of the respondents completing the survey is presented in Table 1 and Table 2. Compared to the national Australian census, the respondents were matched for age and gender, but had a higher percentage of respondents completing Year 12 education or higher, with a weekly household income < $AU650, holding PHI, and having 1 or more household dependents (national census: 43%, 30%, 45%, 12.5% respectively) [30,31]. Just over half (61%) held a health care concession card; 48% were privately insured for health. Most respondents (96%) had experience with taking prescribed medications. Of the respondents currently taking prescribed medications (69%), the majority (96%) had been taking them chronically (>6 months).

**Table 1:** Respondent background characteristics

**Respondent background characteristics**	**Medication users (n = 111)**	**Medication non-users (n = 50)**
Age, median (range), y	59 (21–81)	44 (18–71)*
Gender (male), No. (%)	47 (42%)	25 (50%)
Education, No. (%)		
≤ Year 12 or equivalent	52 (47%)	24 (48%)
> Year 12 or equivalent	59 (53%)	26 (52%)
Income (weekly $AU household, before tax), No. (%)		
< $650	47 (42%)	12 (24%)
≥ $650	46 (41%)	27 (54%)
Prefer not to answer	18 (16%)	11 (22%)
Health Care Concession Card Holder (yes), No. (%)	72 (65%)	26 (52%)
Private Health Insurance (yes), No. (%)	57 (51%)	20 (40%)
Number Household Dependents, No. (%)		
0	86 (77%)	32 (64%)
≥ 1	25 (23%)	18 (36%)
Ever Taken Prescribed Medications (no), No. (%)	0 (0%)	6 (12%)*
Complementary/Natural Therapy Use (yes), No. (%)	54 (49%)	23 (46%)
Beliefs about Medication – general, mean (sd)	26.9 (5.8)	24.6 (5.4)*
Number of prescribed medications, median (range)	3 (0–24)	Not Applicable

^*^ P<0.05 Medication users compared to non-users, χ2 test (proportions), Mann–Whitney U test (non-parametric), independent sample t-test (parametric).

**Table 2:** Current prescribed medication use

**Current prescribed medication use**		**Medication users (n = 111)**
>6 months duration of use, (yes), No. (%)		106 (96%)
7-day non-adherence (yes), No. (%)		24 (22%)
12-month non-adherence, (yes), due to:		
Cost No. (%)		8 (7%)
Side Effects, No. (%)		9 (8%)
Other, No. (%)		16 (14%)
12-month non-persistence (yes), due to:		
Cost, No. (%)		12 (11%)
Side Effects, No. (%)		15 (14%)
Other, No. (%)		14 (13%)

### Validity of responses

Of the 161 responses, 91% passed the monotonicity test generally indicating “rational” interpretation of the factors as intended. Additionally, most of the parameter estimates were significant (*P* < 0.05) and, with the exception of symptom severity, were in the expected direction. For the repeated choice task, 77% of the respondents passed and the kappa statistic was 0.48 representing moderate agreement [32]. Two separate models excluding respondents who had failed each test were run to investigate the effects on the model. For the monotonicity test, there was no difference in model parameter estimates. For the completeness tests, the loss of statistical power did not change the relative magnitude between parameter estimates. As the deletion of such responses has recently been cautioned against due in part to the shortcomings of the tests to truly detect irrationality and consequent removal of potentially valid preference responses, and considering that the existence of such preferences can be seen to be consistent with random utility theory [24,33], all responses were included in the final model.

### DCE results

Additional file 2 shows the results of the DCE. The results of the final model are displayed. The β coefficients represent the relative impact of a unit change of each factor on the preference to continue with a medication. For instance, the negative sign of the side effect severity parameter indicates that an increase in the side effect severity of a medication decreases the relative preference for continuing with a medication.

Six of the eight factors were found to have a statistically significant effect on the decision to continue with a medication. Specifically, those factors were those concerning the harms or benefits of the medication as well as cost and regimen. The presence of statistically significant standard deviations associated with the mean parameter estimates for factors concerning harms and benefits suggest the existence of preference heterogeneity for these factors. The parameter estimates for the severity of daily symptoms and the ability to drink alcohol whilst taking the medication were not found to have a statistically significant influence on respondent preferences for continuing a medication. The constant term (α) was not significant indicating no general preference for Medication ‘A’ over Medication ‘B’ when all factors and levels were the same, and that factors not included in this experiment had no significant effect on the results.

No improvement in the model fit statistics (AIC or McFadden’s pseudo R^2^) occurred with the addition of respondent background characteristics displayed in Table 1 as covariates, including current use of prescribed medications. Additionally, the beta-parameters associated with these covariates were not statistically significant. However, the relative influence of cost on decisions appeared to be influenced by private health insurance status, but not by income or health care concession card status. Specifically, respondents with private health insurance appeared to be less sensitive to cost when deciding to continue with a medication compared to those without private health insurance (β: 0.004, P: 0.0534). As this effect was approaching significance, two separate cost parameters were therefore created for each private health insurance group and incorporated into the model.

For respondents with private health insurance, the out of pocket costs for a medication was not statistically significant (*P* = 0.08). In contrast, for respondents without private health insurance, an increase in the out of pocket cost for a medication was found to decrease the preference to continue with a medication (*P* < 0.001). The final model, displayed in Additional file 2, was found to be statistically significant compared to a model assuming equal choice shares at the α=0.05 level (χ^2^ = 291.1*, df =13). This model represented an acceptable fit [27] and was predicting 58% of actual choices made.

Inputting the parameter estimates from the final model and levels of factors into the utility function (Equation 1) provides information on how respondents were willing to trade between levels of factors. For instance, switching from a medication taken four times a day to one taken once a day, results in an increase in preference (U) to continue with a medication by 0.2811 if all other factor levels remain equal. However, respondents would prefer to continue with the more complex regimen if there was a 20% decrease in the risk of future unwanted medication effects with the new medication relative to the old medication.

The relative importance of the factors using the population beta-estimates and incorporating the range of factor levels is displayed in Additional file 2. In general the ability of the medication to reduce the risk of death was most important, followed by current side effect severity and risk of future side effects. The number of doses per day was of least importance relative to the other significant factors. Accounting for preference heterogeneity around the factors for harms and benefits, using the individual beta estimates to calculate relative importance at the individual level revealed that 58% of respondents considered factors concerning harms of greater importance than benefits (Table 3). Furthermore, with respect to side effects, current side effects were mostly considered as more important than the risk of future side effects. In contrast, the ability of the medication to reduce the risk of death was considered more important than reductions in symptom frequency.

**Table 3:** **Relative importance of medication harms and benefits**^**a**^**- individual level**

**Comparison**	**Number of respondents (%), (n = 161)**
Harms (overall) > Benefits (overall)^b^	94 (58%)
Harms (immediate) > Harms (long-term)^c^	86 (53%)
Benefits (immediate) > Benefits (long-term)^d^	30 (19%)

^a^ Using individual parameter estimates, the relative importance of factors for medication harms and benefits was investigated using the coefficient range for each factor. The coefficient range is the difference between the smallest (negative) part worth utility and the largest part worth utility within factor levels.

^b^ This represents the number of respondents with the sum of the coefficient ranges for the factors “severity of current side effects” and “chance of future unwanted medication effects” greater than the sum of the coefficient ranges for the factors “frequency of symptoms while on the medication (current)” and “chance of early death from the illness while on the medication (future)”.

^c^ This represents the number of respondents with the coefficient range for the factor “severity of current side effects” greater than the coefficient range for the factor “chance of future unwanted medication effects”.

^d^ This represents the number of respondents with the coefficient range for the factor “symptom frequency while on medication (current)” greater than the coefficient range for the factor “chance of early death from the illness while on the medication (future)”.

## Discussion

To the best of our knowledge, this is the first study that, within a community sample, has used a DCE to explore the relative influence of factors on decisions to continue with prescribed medications. Such decisions underscore the concept of intentional medication adherence. The results of this study show that respondents’ preferences to persist with medications were, in order of importance, influenced by long-term medication benefits, current severity and future risk of medication side effects, reduced out-of-pocket costs, short-term medication benefits, and fewer doses per day. This study extends the present literature by characterising the complexity of medication-taking decisions, and in particular, how these decisions are arrived at through the deliberative processes of trading between multiple treatment factors.

Within this study, preferences to continue with the hypothetical medications presented were found to be independent of patient background characteristics previously found to have mixed effects on actual adherence within the literature [26]. This finding suggests that medication-taking decisions are driven by one’s valuation of the underlying attributes of a medication [11] and that, consistent with previous research [13], intentional medication adherence may be a behaviour that is not strongly associated with patient characteristics. This underscores the potential importance of eliciting a patients’ valuation of medication factors as a means to understanding medication-taking decisions.

Consistent with the literature, this study has found that the number of daily medication doses was significant in influencing one’s preference to continue with a medication [26]. However, in contrast to a recent qualitative study [21], the medication-taking decisions in this study were not dominated by one medication factor. By inputting parameter estimates and factor levels, it was shown that reducing the dosing frequency alone might not increase a patient’s decision to continue with a medication if other significant factors, such as the side effect profile of the medication, are perceived as overly compromised. This finding may help to explain why reductions in dosage frequency, as a stand-alone adherence enhancing intervention, have resulted in only modest improvements in overall adherence [5].

Medication cost is generally accepted internationally as a major determinant of adherence [1,34,35]. Within the DCE, when considered alongside other factors, out-of pocket drug cost was found to have a significant influence on one’s preference to continue with a medication. This effect was irrespective of respondent’s income level or if the actual co-payment currently paid for medications was lower. However, out of pocket cost appeared to have a stronger influence on medication choice for those without private health insurance, which is not surprising given that insurance status in Australia may provide supplementary coverage for drugs not covered through the government subsidised social health insurance, Medicare.

The amount, type, and format of information to present to patients, particularly regarding medication harms and benefits, and the effect it has on medication acceptance and behaviour is often debated [36-41]. Alongside other factors, respondents in this study generally took into account both medication harms and benefits into their medication-taking decisions. On the whole, these were considered of higher relative importance than regimen, which is typically a focus for improving adherence. Consistent with the literature [11,42], medication harms were collectively considered of greater importance than medication benefits. The higher relative weight of medication harms and benefits in this study confirms the importance of this information upon medication-taking decisions [36,41] and suggests a need to reflect these preferences in future policies aimed at improving the safe and efficacious use of prescribed medications within the community.

In contrast to what might be expected, the severity of disease symptoms did not have a significant influence on the decision to continue with a medication. This does not imply that symptom severity is not important to the decisions made, but on balance was neither having a positive or negative influence [15,26,31]. Nonetheless, respondents conflating symptom severity with medication side-effects may have influenced this result. Previous research has indicated the difficulties patients have in attributing symptoms to the disease or the medication [11], and this current result may have been influenced by these two factors being described on the same scale. However, a previous DCE of patient preferences for asthma medication has also found a non-significant influence from the combined factor of symptom severity and frequency on medication choice unless reduced to a minimal level [43]. The present study suggests that adverse health outcomes that are believed to be iatrogenic weigh more heavily on patient medication-taking decisions than health outcomes that are associated with the natural course of illness.

The relative importance of immediate versus future health outcomes differed substantially when framed as either medication harms as opposed to therapeutic benefits. Specifically, more respondents placed a greater value on current harms (i.e.: side effect severity) than future harms (i.e. 10-year risk of future side effects) reflecting an implicit discounting of harms. Such reasoning is consistent with conventional economic logic and reflects at an intuitive level the prominence placed on medication side effects on individuals’ day to day experience with medications [11]. However when considering therapeutic benefits the findings are less intuitive. More respondents considered future benefits (i.e. 10-year reduced risk of death) more important than current benefits (i.e. reduced frequency of symptoms), which would suggest a negative rate of discounting for therapeutic benefits. This apparently irrational response could reflect the difficulties respondents may have had discussed above in disentangling possible therapeutic outcomes from changes in health caused by the natural course of illness.

The results of this study must be viewed in light of its limitations. First, the use of an online panel provider has precluded description of non-responders and potential responder bias. In particular, the individuals’ motivational traits, recently demonstrated to predict internet panel and survey participation, may limit the generalisability from web-panels based on strictly demographics-based weighting schemes [44]. Additionally, although an MMNL model was used to account for the unobservable preference heterogeneity in the sampled population, other background medical information, particularly the number, type and experience with chronic conditions and medications, including psychiatric conditions, as well as cultural/ethnicity information were not accounted for. Furthermore, the sample was not matched to the national Australian census on other potentially important variables such as education and income. It is also acknowledged that this study has been conducted within one health system. These factors may limit the generalisability of the findings. However, this study was not attempting to quantify preferences or generalise such numbers, but rather, investigate the relationship between factors relevant to a variety of medications that may be used across many different conditions and health care settings. The lack of difference in patient preferences between different health care settings found in another study [20], particularly considering cost was not the primary driver within our study, supports the international relevance of this study. Nonetheless, although the use of an efficient design facilitates smaller sample sizes [45], larger, international, studies across different healthcare settings and amongst subsets of chronically ill patients and medication users, that are statistically powered for *a priori* subgroup analyses, are warranted.

Second, respondents were given generic descriptions of disease and treatment. Whilst it could be argued that there are significant challenges in responding to this type of abstraction, it was felt that this was necessary to mimic decisions across multiple diseases and medications and thus address the main study question. Encouragingly, a meta-analysis of non-psychiatric adherence research over 50 years and across many different conditions including HIV, cardiovascular disease, and neurological disorders, has found that non-adherence was not statistically significantly related to the type or severity of disease [46].

Finally, while discrete choice methods are widely used in health economics, an inherent limitation is that respondents are evaluating hypothetical medications; that is what respondents declare they will do may be quite different to what they would actually do when they experience the consequences of a choice [27]. Arguably, forcing respondents to choose between medications may also be contrary to actual behaviour [25], particularly considering the over-riding influence of a prescribers’ recommendation upon patient preventive treatment decisions suggested by Gale et al [47]. To minimise such potential differences, measures were taken to design the hypothetical tasks to be as realistic as possible [15,27], for instance by centring levels of cost about current medication co-payments and describing severity of medication harms and benefits in terms of their effect on health status. Encouragingly, a recent study that applied the discrete choice framework to analyse actual adherence data from a HIV clinical trial dataset has also found side effects, regimen and medication effectiveness to influence adherence [48]. As this study has measured intended medication-taking decisions, future studies incorporating actual adherence data [27,48] are needed to determine the relationship between intended and actual behaviour.

## Conclusions

Improving adherence to medications, particularly for chronic conditions, has been highlighted as a priority area for policy development [49] and for improving clinical practice [50]. To do so, it must be acknowledged that, outside of the consultation room, patients ultimately have and do exercise the power to follow, modify or reject prescribed treatment [11]. That is, a part of this health behaviour is the subject of rational choices, influenced by the attributes of treatments and potentially amenable to intervention through education, strategic pricing and the altering of dosing characteristics. Understanding individual treatment preferences is thus an important step to improving adherence support provision in practice. Considering the modest effect of current adherence-enhancing interventions to date [5,6], re-framing future interventions and policies to acknowledge and support rational and informed individual patient decisions that may underpin intentional medication adherence behaviour, rather than simply addressing ‘aberrant’ and ‘irrational’ behaviour, may be the way forward to realising the full potential health and economic benefits from the efficacious use of medications.

## Competing interests

The authors declare they have no competing interests.

## Authors’ contributions

This work is a component of TL’s doctoral research supervised by SJ and JB. Thus TL, SJ and JB have all made substantial contributions to the conception, design, acquisition, analysis and interpretation of the data, as well to the critical revision of the manuscript. All authors were involved in initial conception of the paper and in the design of the study. TL developed the survey, conducted the analysis and wrote the first draft. All authors contributed to the preparation of the final manuscript. All authors have read and approved the final manuscript.

## Pre-publication history

The pre-publication history for this paper can be accessed here:

http://www.biomedcentral.com/1471-2296/13/61/prepub

## Supplementary Material

Additional file 1 **Description of factors and levels used in the Discrete Choice Experiment [**[51]**,**[52]**].** Click here for file

Additional file 2 **Discrete choice experiment results [**[17]**,**[28]**].** Click here for file

## References

[B1] Sabate E Adherence to long term therapies: Evidence for action 2003 World Health Organisation, Geneva, Switzerland available at http://www.who.int/chronic_conditions/adherencereport/en/

[B2] Humphrey K Weeramanthri T Fitz J Forgetting Compliance 2001 University Press, Northern Territory

[B3] Osterberg L Blaschke T Adherence to medication N Engl J Med 2005 353 5 487 497 10.1056/NEJMra050100 16079372

[B4] Elliott RA Barber N Horne R Cost-effectiveness of adherence-enhancing interventions: a quality assessment of the evidence Ann Pharmacother 2005 39 3 508 515 10.1345/aph.1E398 15657115

[B5] Haynes RB Ackloo E Sahota N McDonald HP Yao X Interventions for enhancing medication adherence.[update of Cochrane Database Syst Rev. 2005;(4):CD000011; PMID: 16235271] Cochrane Database Syst Rev 2008 2 CD000011 18425859 10.1002/14651858.CD000011.pub3

[B6] Schedlbauer A Davies P Fahey T Interventions to improve adherence to lipid lowering medication Cochrane Database Syst Rev 2010 3 CD004371 20238331 10.1002/14651858.CD004371.pub3

[B7] Cleemput I Kesteloot K DeGeest S A review of the literature on the economics of noncompliance. Room for methodological improvement Health Policy 2002 59 1 65 94 10.1016/S0168-8510(01)00178-6 11786175

[B8] Krigsman K Nilsson JLG Ring L Adherence to multiple drug therapies: refill adherence to concomitant use of diabetes and asthma/COPD medication Pharmacoepidemiol Drug Saf 2007 16 10 1120 1128 10.1002/pds.1433 17566142

[B9] Lehane E McCarthy G Intentional and unintentional medication non-adherence: a comprehensive framework for clinical research and practice? A discussion paper Int J Nurs Stud 2007 44 8 1468 1477 10.1016/j.ijnurstu.2006.07.010 16973166

[B10] Lehane E McCarthy G An examination of the intentional and unintentional aspects of medication non-adherence in patients diagnosed with hypertension J Clin Nurs 2007 16 4 698 706 10.1111/j.1365-2702.2005.01538.x 17402951

[B11] Pound P Britten N Morgan M Yardley L Pope C Daker-White G Campbell R Resisting medicines: A synthesis of qualitative studies of medicine taking Social Science & Medicine 2005 61 1 133 155 10.1016/j.socscimed.2004.11.063 15847968

[B12] Stack RA Elliott R Noyce PR Bundy C A qualitative exploration of multiple medicines beliefs in co-morbid diabetes and cardiovascular disease Diabetic Medicine 2008 25 1204 1210 10.1111/j.1464-5491.2008.02561.x 19046199

[B13] Wroe AL Intentional and unintentional nonadherence: a study of decision making J Behav Med 2002 25 4 355 372 10.1023/A:1015866415552 12136497

[B14] Wu JR Moser DK Lennie TA Peden AR Chen YC Heo S Factors influencing medication adherence in patients with heart failure Heart and Lung: Journal of Acute and Critical Care 2008 37 1 8 16 e11 10.1016/j.hrtlng.2007.02.003 18206522

[B15] Lancsar E Louviere J Conducting discrete choice experiments to inform healthcare decision making: a user’s guide Pharmacoeconomics 2008 26 8 661 677 10.2165/00019053-200826080-00004 18620460

[B16] Louviere JJ Lancsar E Choice experiments in health: the good, the bad, the ugly and toward a brighter future Health Econ Policy Law 2009 4 Pt 4 527 546 19715635 10.1017/S1744133109990193

[B17] Ryan M A role for conjoint analysis in technology assessment in health care? Int J Technol Assess Health Care 1999 15 3 443 457 10874373

[B18] Ryan M Gerard K Using discrete choice experiments to value health care programmes: current practice and future research reflections Appl Health Econ Health Policy 2003 2 1 55 64 14619274

[B19] Johnson FR Ozdemir S Manjunath R Hauber AB Burch SP Thompson TR Factors that affect adherence to bipolar disorder treatments: a stated-preference approach Med Care 2007 45 6 545 552 10.1097/MLR.0b013e318040ad90 17515782

[B20] Hauber AB Mohamed AF Johnson FR Falvey H Treatment preferences and medication adherence of people with Type 2 diabetes using oral glucose-lowering agents Diabet Med 2009 26 4 416 424 10.1111/j.1464-5491.2009.02696.x 19388973

[B21] Elliott RA Ross-Degnan D Adams AS Safran DG Soumerai SB Strategies for coping in a complex world: adherence behavior among older adults with chronic illness J Gen Intern Med 2007 22 6 805 810 10.1007/s11606-007-0193-5 17406952 PMC2219857

[B22] Horne R Weinman J Hankins M The beliefs about medicines questionnaire: The development and evaluation of a new method for assessing the cognitive representation of medication Psychology & Health 1999 14 1 1 24 10.1080/08870449908407311

[B23] Ngene 1.0 [http://www.choice-metrics.com/]

[B24] Lancsar E Louviere J Deleting ‘irrational’ responses from discrete choice experiments: a case of investigating or imposing preferences? Health Econ 2006 15 8 797 811 10.1002/hec.1104 16615039

[B25] Louviere J Henscher DA Swait J Stated Choice Methods: Analysis and Application 2000 Cambridge University Press, Cambridge

[B26] Krueger KP Berger BA Felkey B Medication adherence and persistence: a comprehensive review Adv Ther 2005 22 4 313 356 10.1007/BF02850081 16418141

[B27] Hensher DA Rose JM Greene WH Applied Choice Analysis: A Primer 2005 Cambridge University Press, UK

[B28] Kimman ML Dellaert BG Boersma LJ Lambin P Dirksen CD Follow-up after treatment for breast cancer: one strategy fits all? An investigation of patient preferences using a discrete choice experiment Acta Oncol 2010 49 3 328 337 10.3109/02841860903536002 20148645

[B29] The American Association for Public Opinion Research Standard Definitions: Final Dispositions of Case Codes and Outcome Rates for Surveys 2011 7 AAPOR

[B30] AIHW Australia’s Health 2010. Australia’s health no. 12. Cat no. AUS122 2010 AIHW, Canberra

[B31] Australian Bureau of Statistics 2006, 2006 Census of Population and Housing, Gross family income (weekly) by family composition; Highest Year of School completed by Age and Sex for persons no longer at school; Family composition and Social Marital Status by number of dependent children for time series data cube: Excel spreadsheet, cat. no. 2068.0, viewed 16 April 2010, http://www.censusdata.abs.gov.au

[B32] Landis JR Koch GG The measurement of observer agreement for categorical data Biometrics 1977 33 1 159 174 10.2307/2529310 843571

[B33] Ryan M Watson V Entwistle V Rationalising the 'irrational': a think aloud study of discrete choice experiment responses Health Econ 2008 31 3 321 336 10.1002/hec.1369 18651601

[B34] Briesacher BA Gurwitz JH Soumerai SB Patients at-risk for cost-related medication nonadherence: a review of the literature J Gen Intern Med 2007 22 6 864 871 10.1007/s11606-007-0180-x 17410403 PMC2219866

[B35] Ingersoll KS Cohen J The impact of medication regimen factors on adherence to chronic treatment: a review of literature J Behav Med 2008 31 3 213 224 10.1007/s10865-007-9147-y 18202907 PMC2868342

[B36] Crockett RA Sutton S Walter FM Clinch M Marteau TM Benson J Impact on Decisions to Start or Continue Medicines of Providing Information to Patients about Possible Benefits and/or Harms: A Systematic Review and Meta-Analysis Med Decis Making 2011 31 5 767 777 10.1177/0272989X11400420 21447731

[B37] Stevenson FA Cox K Britten N Dundar Y A systematic review of the research on communication between patients and health care professionals about medicines: the consequences for concordance Health Expectations 2004 7 3 235 245 10.1111/j.1369-7625.2004.00281.x 15327462 PMC5060245

[B38] Hembroff LA Holmes-Rovner M Wills CE Treatment decision-making and the form of risk communication: results of a factorial survey BMC Med Inf Decis Mak 2004 4 20 10.1186/1472-6947-4-20 PMC535806 15546488

[B39] Peters E Hart PS Fraenkel L Informing patients: the influence of numeracy, framing, and format of side effect information on risk perceptions Medical Decision Making 2011 31 3 432 436 10.1177/0272989X10391672 21191122

[B40] Short D Frischer M Bashford J Ashcroft D Why are eligible patients not prescribed aspirin in primary care? A qualitative study indicating measures for improvement BMC Fam Pract 2003 4 9 10.1186/1471-2296-4-9 12871601 PMC183829

[B41] Young SD Oppenheimer DM Different methods of presenting risk information and their influence on medication compliance intentions: results of three studies Clin Ther 2006 28 1 129 139 10.1016/j.clinthera.2006.01.013 16490587

[B42] Clifford S Barber N Horne R Understanding different beliefs held by adherers, unintentional nonadherers, and intentional nonadherers: application of the Necessity-Concerns Framework Journal of Psychosomatic Research 2008 64 1 41 46 10.1016/j.jpsychores.2007.05.004 18157998

[B43] King MT Hall J Lancsar E Fiebig D Hossain I Louviere J Reddel HK Jenkins CR Patient preferences for managing asthma: Results from a discrete choice experiment Health Econ 2007 16 7 703 717 10.1002/hec.1193 17238221

[B44] Bruggen E Dholakia UM Determinants of Participation and Response Effort in Web Panel Surveys Journal of Interactive Marketing 2010 24 239 250 10.1016/j.intmar.2010.04.004

[B45] Rose J Bliemer MCJ Constructing efficient choice experiments. Report ITLS-WP-05-07 2005 Institute of Transport and Logistics Studies, University of Sydney

[B46] DiMatteo MR Variations in patients’ adherence to medical recommendations: a quantitative review of 50 years of research Medical Care 2004 42 3 200 209 10.1097/01.mlr.0000114908.90348.f9 15076819

[B47] Gale N Greenfield S Gill P Gutridge K Marshall T Patient and general practitioner attitudes to taking medication to prevent cardiovascular disease after receiving detailed information on risks and benefits of treatment: a qualitative study BMC Fam Pract 2011 12 1 59 10.1186/1471-2296-12-59 21703010 PMC3135546

[B48] Lamiraud K Geoffard P-Y Therapeutic non-adherence: a rational behavior revealing patient preferences? Health Econ 2007 16 11 1185 1204 10.1002/hec.1214 17304501

[B49] Mitka M Improving medication adherence promises great payback, but poses tough challenge JAMA 2010 303 9 825 10.1001/jama.2010.212 20197524

[B50] Baker R King’s Fund report on improving the quality of care in general practice BMJ 2011 342 d1932 10.1136/bmj.d1932 21436117

[B51] Elwyn G O’Connor A Stacey D Volk R Edwards A Coulter A Thomson R Barratt A Barry M Bernstein S Developing a quality criteria framework for patient decision aids: online international Delphi consensus process BMJ 2006 333 7565 417 10.1136/bmj.38926.629329.AE 16908462 PMC1553508

[B52] Griffith JM Lewis CL Hawley S Sheridan SL Pignone MP Randomized trial of presenting absolute v. relative risk reduction in the elicitation of patient values for heart disease prevention with conjoint analysis Medical Decision Making 2009 29 2 167 174 19279298 10.1177/0272989X08327492

