# Supplementary material for: Understanding rational non-adherence to medications. A discrete choice experiment in a community sample in Australia
Source: BMC Fam Pract. 2012 Jun 20;13:61. doi: 10.1186/1471-2296-13-61 (PMC3511288; doi:10.1186/1471-2296-13-61)
Supplement: Additional file 1 — Description of factors and levels used in the Discrete Choice Experiment [[51],[52]]. [file 1471-2296-13-61-S1.pdf]

Additional File 1 - Description of factors and levels used in the Discrete Choice Experiment

**Factor**		**Description**	**Levels**[a]
Medication Harms			
Immediate	Severity of **current** side effects	The severity of daily medication side effects is:	1, 4, 7, 10 (out of 10)[b]
Long-term	Chance of **future** unwanted medication effects	For every 100 people taking this medication, the number of people who will have unwanted effects in the next 10 years is (this may or may not be you):	5, 35, 65, 95[c]
Medication Benefits			
Immediate	Symptom frequency while on the medication **(current)**	On medication, daily symptoms are now felt:	2, 3, 5, 6 (days/week)
Long-term	The chance of early death from the illness while on the medication (**future)**	For every 100 people taking this medication, 85 will die in the next 10 years. This may or may not be you. On medication, this number reduces to:	5, 25, 45, 65[c]
Cost		Your monthly cost for this medication:	$AUS 0, 10, 35, 50[d]
Symptom Severity		Symptom severity is	1, 4, 7, 10 (out of 10)[b]
Medication Regimen		The medication is taken:	1, 2, 3, 4 (times/day)
Alcohol Restrictions		Can you drink alcohol while on this medication?	Yes, No (Base)

a *A priori* expectations were that respondents would prefer the alternative with lower side effect severity, risk of future unwanted medication effects, symptom frequency, risk of death, cost and regimen. No a priori expectations were made regarding symptom severity or alcohol restrictions.

b The severity of side effects and symptoms were rated on a common scale and described in terms of the effect on one's ability to perform daily activities

c Developed and explained with reference to the International Patient Decision Aids Standards Collaboration (IPDAS) background document [51] and in light of current research surrounding risk presentation in preference elicitation techniques [52].

d Levels were centred about the current Australian Pharmaceutical Benefits Schedule general and concession co-payment for 2010 for prescribed medication
