# Supplementary material for: Understanding rational non-adherence to medications. A discrete choice experiment in a community sample in Australia
Source: BMC Fam Pract. 2012 Jun 20;13:61. doi: 10.1186/1471-2296-13-61 (PMC3511288; doi:10.1186/1471-2296-13-61)
Supplement: Additional file 2 — Discrete choice experiment results [[17],[28]]. [file 1471-2296-13-61-S2.pdf]

Additional File 2 - Discrete Choice Experiment results

Factor			β Estimate	Standard Error	Lower 95% CI	Upper 95% CI	P	Relative Importance[a]
**Medication Harms[b]**								
Immediate	Severity of current side effects	Mean	-0.1569	0.0199	-0.1960	-0.1178	<0.001	0.242 (2)
		SD	0.1234	0.0262	0.0719	0.1748	<0.001	
Long-term	Chance of future unwanted medication effects	Mean	-0.0140	0.0021	-0.0181	-0.0099	<0.001	0.216 (3)
		SD	0.0134	0.0030	0.0075	0.0192	<0.001	
**Medication Benefits[b]**								
Immediate	Symptom frequency while on medication (current)	Mean	-0.1451	0.0336	-0.2110	-0.0792	<0.001	0.100 (5)
		SD	0.1764	0.0609	0.0570	0.2958	<0.01	
Long-term	Chance of early death from illness while on the medication (future)	Mean	-0.0301	0.0039	-0.0378	-0.0223	<0.001	0.309 (1)
		SD	0.0305	0.0046	0.0215	0.0395	<0.001	
**Cost[c]**	With Private Health Insurance	Mean	-0.0064	0.0037	-0.0136	0.0008	0.08	
	Without Private Health Insurance	Mean	-0.0151	0.0036	-0.0222	-0.0080	<0.001	0.130 (4)
**Symptom Severity[c]**		Mean	-0.0093	0.0149	-0.0384	0.0199	0.53	
**Medication Regimen[c]**		Mean	-0.0937	0.0444	-0.1807	-0.0068	0.03	0.048 (6)
**Alcohol Restrictions (Yes)[c]**		Mean	0.0758	0.0893	-0.0992	0.2508	0.40	
**Constant[c]**		Mean	0.0472	0.0779	-0.1055	0.1999	0.54	
**Model Fit Statistics**								
	Log Likelihood				-746.2			
	McFadden's ρ^2 adjusted				0.1542			
	Akaike's Information Criterion				1.18			

a The relative importance of factors and their levels was determined by calculating the coefficient range, which is the difference between the smallest (negative) part worth utility and the largest part-worth utility within the factor levels, and dividing it by the sum of the coefficient ranges for all factors and levels [17, 28]

b Random parameter

c Non-random parameter
